# Emerging role of mTOR in tumor immune contexture: Impact on chemokine-related immune cells migration

**DOI:** 10.7150/thno.45219

**Published:** 2020-05-15

**Authors:** Jing Jin, Qijie Zhao

**Affiliations:** 1 Department of Oncology, The Affiliated Hospital of Southwest Medical University, Luzhou, Sichuan 646000, P.R. China; 2 Laboratory of Molecular Pharmacology, Southwest Medical University, Luzhou, 646000, Sichuan, PR China; 3 Department of Pathophysiology, College of Basic Medical Science, Southwest Medical University, Luzhou, 646000, Sichuan, PR China; 4 South Sichuan Institute of Translational Medicine, Luzhou, 646000, Sichuan, PR China

**Keywords:** mTOR, Chemokine, Chemotaxis, Immune cells, Tumor microenvironment (TME)

## Abstract

During the last few decades, cell-based anti-tumor immunotherapy emerged and it has provided us with a large amount of knowledge. Upon chemokines recognition, immune cells undergo rapid trafficking and activation in disease milieu, with immune cells chemotaxis being accompanied by activation of diverse intercellular signal transduction pathways. The outcome of chemokines-mediated immune cells chemotaxis interacts with the cue of mammalian target of rapamycin (mTOR) in the tumor microenvironment (TME). Indeed, the mTOR cascade in immune cells involves migration and infiltration. In this review, we summarize the available mTOR-related chemokines, as well as the characterized upstream regulators and downstream targets in immune cells chemotaxis and assign potential underlying mechanisms in each evaluated chemokine. Specifically, we focus on the involvement of mTOR in chemokine-mediated immune related cells in the balance between tumor immunity and malignancy.

## Background

Cancer is a life-threatening disease traditionally categorized by cells and tissue types based on origins. With advance technology of sequencing methodologies and carcinogenic mechanisms, we now understand that considerable genomic, transcriptomic, and epigenetic variation exist within various tumor types. This, in turn, has led to improvement in therapeutic strategies for some patients, such as estimating the response to targeted and individualized therapies for patients based on stratified cancer molecular characteristics 1. Rather than the “one dose suits all” approach, genomic analysis as a methodology aims to target novel disordered biological targets in tumor for individualized treatment 2. More recently, with high-throughput tumor sequencing, immune cell populations were found to continuously enrich in tumor microenvironment (TME) and constituted a vital element of tumor tissues 1, 3, 4. Indeed, cancer is observably facilitated by immune system disorder, and immune cells play an important role in TME and shape the hallmark of heterogeneous cancer cells survival and resistance to therapy 5. Increasing body of evidence demonstrated that TME is significantly affected by misled or diminished immune cells responses, such as gastric, liver, lung, melanoma, and breast cancer 1, 3, 4, 6, 7. Immune cells accumulation or loss in TME is important for tumorigenesis or malignancy, but the underlying mechanisms are still unclear 3, 8. Now, with multiple approaches in investigation, tumor immune cells exert their capacity to cooperate with appropriate adaptive signaling cascades in response to immunological stimuli 9, 10.

The mammalian target of rapamycin (mTOR), an evolutionarily conserved serine/threonine kinase, is mostly involved in the central immune microenvironment to regulate cellular functions such as growth, proliferation and survival 11, 12. Two mTOR protein complexes (mTORC1 and mTORC2) 13, 14, defined by the association of mTOR with the adaptor proteins Raptor and Rictor, have been proved to act as the central nodes of the phosphoinositide 3-kinase (PI3K)/AKT downstream signaling pathway effector 15, 16. mTOR is generally regarded as a potential oncogene in an effective anti-cancer target therapy 11, 17, 18. Dysregulation of different protein complexes (mTORC1 and mTORC2) were proved to be connected with pathological alteration in tumorigenesis 11, 13. Critically, clinical application of mTOR cascade intervention did not achieve satisfactory clinical outcomes due to a variety of reasons 19. Moreover, deregulation of mTOR signaling was found to play a crucial role in regulating the immune responses, such as in T cell and myeloid cell differentiation, and multiple metabolic functions 16, 20. mTOR selective inhibition has a profound effect on immune cell populations, including CD8^+^ T cells, CD4^+^ T cells, CD3^+^ T cells and B cells, and also antitumor immunity 21. In line with this, immune recognition can contribute to tumor suppression, resulting in enhanced cell infiltration and acts as a molecular signature for tumor immune microenvironment activation 22. However, the molecular mechanisms of the immune cell function or migration are only partly understood. The chemokines were reported to not merely regulate immune heterogeneity and immunotherapy sensitivity, but rather shape the TME immune cell populations 22, 23. The chemokines (CXCL9, CXCL10, and CXCL11) have been demonstrated to connect with T helper type 1 (Th1) cells immunity activation in TME and provide a favorable response to immunotherapy 23, 24. Multiplicity of chemokines within tumors may obscure the contributions of individual chemokines mechanism in immune cell chemotaxis, but cascade signaling is indispensable for these processes.

In this review, we discuss the mTOR signaling pathway cascade, focusing on the immune cell chemotaxis and function in human cancers. Current evidence suggests that the mTOR pathway is closely connected with immune cells and chemokines in tumors, but how this mechanism is orchestrated in the TME and the ability of mTOR to conditioning signal is still unclear. The focus of this review is to provide insights for further speculations for potential modulators towards effective immunotherapy.

### mTOR signal in tumor immune microenvironment

**Figure 1:**
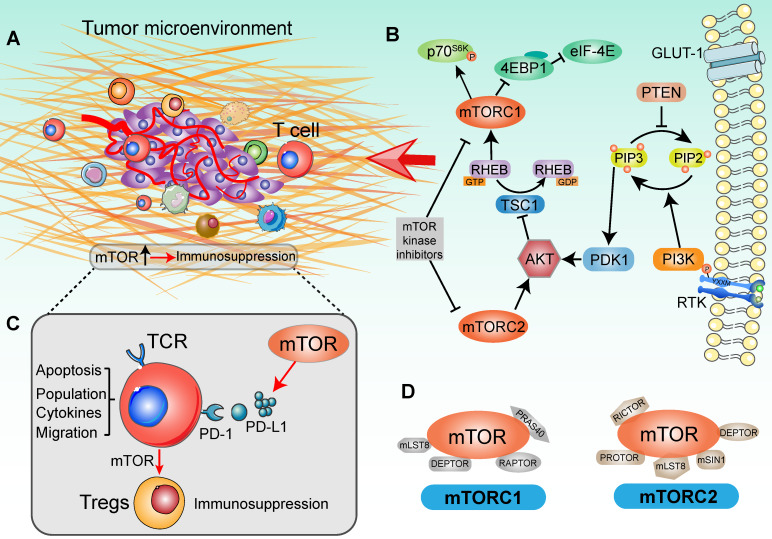
** Signaling cascade with mTOR activation in a tumor immune setting**. **(A)** Tumor immune microenvironment. Tumor anatomy presents tumor foci environmental characteristics, including tumor core and immune environment. (Adapted with permission from 35, copyright 2012 Fridman). **(B)** mTOR-based immune cells strategy was presented in the context of the PI3K/AKT/mTOR signaling network. For the upstream, extracellular cytokines or chemokines stimulation lead to the recruitment of PI3 kinase (PI3K), thereby phosphorylating phosphatidylinositol 4,5-bisphosphate (PIP2) at the 3' position to generate phosphatidylinositol 3,4,5-trisphosphate (PIP3). In this catalytic node, PTEN plays an opposite role to PI3K. Activated PIP3 leads to the recruitment of AKT in signaling cascade by PDK1. Alternatively, mTORC2 is involved in regulating lipid biosynthesis downstream of AKT. AKT activation negatively modulates TSC1, which shapes the GAP activity for RHEB-GTP and increases RHEB-GTP accumulation. This promoted the function of mTORC1. In tumor immune environment, mTORC1 phosphorylates 4EBP1 and S6K to activate critical drivers of global protein translation, inflammation response, cell proliferation and infiltration. Additionally, many immunologic inputs also play a critical role in regulating mTOR signaling activity. Rapamycin and other mTOR inhibitors potently affect both mTOR complexes in an inhibitory manner. **(C)** The function of mTOR in T cell-based immunosuppression. mTOR promotes the PD-L1 expression to recognize the T-cell immune checkpoint, subsequently decreasing the tumor-infiltrating T cells activity. Accompany with the mTOR function, naïve T cells differentiate into Tregs and contribute to immunosuppression. **(D)** Two mTOR signaling complexes with specific substrates. mTOR is associated with two distinct sets of adapter proteins. mTORC1 complex is composed of the mLST8, DEPTOR, RAPTOR and PRAS40; and mTORC2 complex is composed of PROTOR, DEPTOR, RICTOR, mLST8, mSIN1.

The surveillance of TME was proposed to help determine whether an activated immune related pathway promotes or restrains antitumor immunity 25 (Figure 1A). Importantly, individual immunotherapies tailored to optimal pathways have achieved great clinical efficacy in tumor eradication, activation of tumor infiltrating lymphocytes (TILs) and in the function of antigen presenting cells (APCs) 26. Deregulation of mTOR cascade increasingly affects immune effector function and influences the features of the tumor immune microenvironment in human cancers 27 (Figure 1B). The mTOR cascade also shows ability to shape different paracrine and autocrine stimuli in TME and modulates tumor immunity 28. In line with this, based on clinical trials and pre-clinical data, the therapeutic target of the PI3K/AKT/mTOR signaling pathway dose not merely attenuates tumor malignancy and metastasis, but also enhances the tumor immunosurveillance and anti-tumor immunity properties 29. Schematically, in cells growth, disordered growth factors, chemokines or cytokines can stimulate the activation of receptor tyrosine kinase (RTK), the intermediates of which are capable of activating the down-stream PI3K molecules 30. These kinases catalyze the conversion of PIP2 (phosphatidylinositol-4,5-bisphosphate) to PIP3 (phosphatidylinositol-3,4,5-trisphosphate), following which the lipid products recruit pleckstrin homology (PH) domain-containing proteins such as AKT, mTOR and PDK11 31. Ultimately, AKT kinase activates the mTORC1 to promote tumor cell proliferation and protein synthesis 32 (Figure 1D). Generally, hyperactivation of the components of the mTOR pathway contributes significantly to tumorigenesis and malignancy. It thus appears that targeting of the oncogenic mTOR pathway may be a potential cancer therapy strategy 33, 34. However, the therapeutic effects on tumor PI3K-AKT-mTOR pathway network were not always encouraging, which may be due to complex of biological effects in modulating the tumor immune microenvironment 35, 36.

Crosstalk among the immune response and signaling pathways normally ensures an elegant balance between cells function, growth and division 10, 37. TME dysregulation plays a critical role in tumor development because it contains various types of immune cells, cytokines and chemokines, which can either promote or suppress tumorigenesis 35. As an important component of the adaptive immune response, T cells are characterized by their rapidly recall response and signaling from immune microenvironment to dictate the effector response 38. mTOR is emerging as an important modulator in the function of T-cells, macrophages, dendritic cells (DCs), and B cells, and controls multiple metabolic pathways to guide the immune microenvironment, such as glucose uptake, lipid and amino acid metabolism 12, 20, 39. A large number of trials evaluated mTOR inhibitors as modulators to promote anti-tumor immunity and immune cell function (Table 1). Previous studies have indicated that upon inhibition of mTOR signaling, the stimulated T-cell receptor (TCR) could lead to generation of regulatory T cells (Tregs) and proliferation of memory CD8+ T cells 40, 41. Moreover, deletion of the mTORC1 upstream regulator TSC2 and RAS homolog enriched in brain (RHEB) can affect CD8^+^ T cells proliferation and differentiation in antitumor immunity 42. Through small GTPase RHEB function, mTORC1 promotes the T-cell differentiation into the Th1 43. Strikingly, alongside analyses of mTOR selective inhibitor, mTOR cascade has been confirmed as an essential role in T cells differentiation. mTORC1 with metabolic programming is important in T cells activation and T helper 2 (Th2) cells differentiation, which is linked to the glucose metabolism by cytokine responsiveness 44. Recently, in humoral adaptive immunity, T follicular helper (Tfh) cells differentiation was also deemed to depend on mTOR signaling and glucose metabolism 45. The evidence showed that mTORC1 boosted T cells proliferation to meet the cell division needs for Tfh cells differentiation, whereas mTORC2 mainly promoted Tfh cells differentiation by inducing AKT activation and TCF1 expression 46. Meanwhile, with the distinct connection to PKC and AKT cascade in T cells, mTORC2 regulated the differentiation of Th1 and Th2 cells subsets, respectively 47. Complex mTOR signaling mechanisms may co-exist in the different disease conditions 48. These findings define a specific role for mTOR signaling as a vital integrator of diverse immune cells functions in the tumor immune microenvironment.

Oncogenic activation of the mTOR signal pathway is involved in promoting immune escape by driving the expression of PD-L1, subsequently decreasing the concentration of tumor-infiltrating T cells 49, 50 (Figure 1C). After the PD-L1 recognizes T cells receptor PD-1, tumor primary T-cell apoptosis is significantly higher than normal tissues 51. Although there are multiple mechanisms that contribute to tumor immune function, PD-L1 plays a prominent role in many tumors 52, 53. In a mouse model of lung cancer, PD-L1 protein expression mainly depends on activated mTOR cascade, rather than specific oncogene or cytokine stimuli 49. Furthermore, along with the T cells mTOR signaling pathway activation, PD-1 and PD-L1 interaction induced differentiation of naïve CD4^+^ T cells into Tregs and maintained immunosuppressive functions 54. Along with these lines, elevated intracellular mTOR signaling and PD-L1 expression influenced the immune cell populations in TME 55. Conversely, additive effects of anti-PD-L1 and mTOR intervention therapies further promoted the distribution of CD8^+^ T cells and IFN-γ production capacity in TME, with more T cells being infiltrated and activated after targeted immunotherapy 56. Taken together, mTOR is not only involved in tumor progression, but also influences the biological environment of immune cells.

### mTOR and chemokines-mediated immune cells chemotaxis

**Figure 2:**
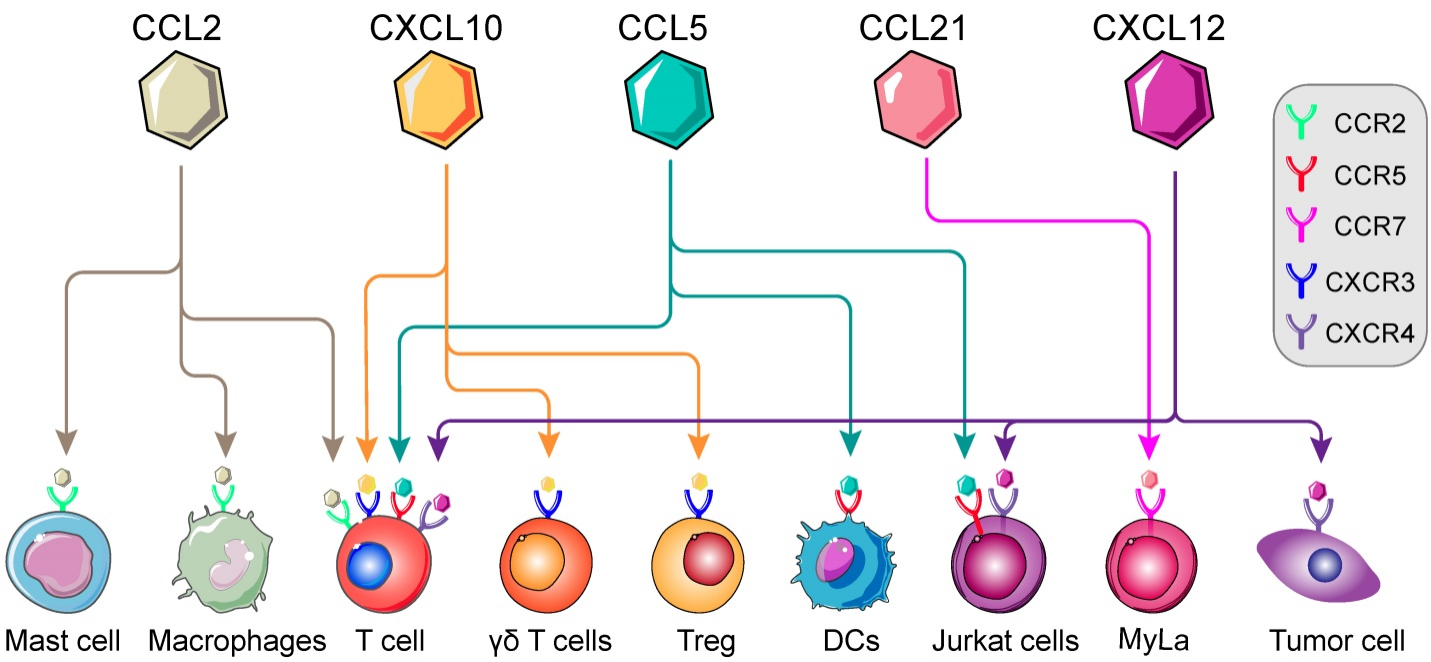
** Landscape of mTOR-related chemokines in immune cells**. Immunological regulation between immune cells, cytokines and chemokines are potentially targetable in TME. The recognition and interaction of chemokines by corresponding receptors are essential for the chemotaxis of immune cells, namely T cells, Regulatory T cells (Tregs), macrophages, Dendritic cells (DCs), Mycosis fungoides cells (Myla cells); Mast cells and so on. (Adapted with permission from 35, copyright 2012 Fridman)

In previous studies, immune cells intracellular mTOR cascade was suggested to modulate multiple immune cells, namely T cells, macrophages, Th1 and Tregs 41, 43, 57. mTOR intervention could alter the types of immune cell populations in various conditions 21, 58. Moreover, involvement of mTOR cascade in chemokine-mediated immune cells chemotaxis has recently been shown in different tumor types 59-61 (Figure 2). Thus, in TME, we postulated that chemokine-induced immune cells chemotaxis is on an mTOR-dependent manner and invokes multistep signaling in target cells. The chemotaxis-related mTOR cascade might be a potential target for anti-tumor immunotherapy 62. In this review, we will investigate the involvement of mTOR-related chemokines (CCL2, CCL5, CCL21, CXCL10 and CXCL12) in immune cells chemotaxis, with particular emphasis on the role of mTOR (Table 2).

### CCR2/CCL2 signal

CCR2, the only known receptor for chemokine C-C motif ligand 2 (CCL2), was expressed on monocytes and macrophages 63. Moreover, CCL2 is known as monocyte chemoattractant protein-1, plays an important role in the recruitment of cancer related monocytes, such as macrophages 64. Takanori et al. indicated that activated CCL2 and its receptor CCR2 can trigger the chemokine signaling in macrophages and promote cancer cells metastasis 65. Furthermore, in colorectal cancer, mTORC2 activity in tumor-associated macrophages (TAMs) was correlated with tumorigenesis 66. Macrophages can alter their function in response to the local microenvironment and cause injury or repair effects. However, the signal required to switch macrophages into positive or negative roles remains poorly understood 67. Accumulating evidence has demonstrated that CCL2 orchestrates the recruitment of TAMs in TME and is a positive sign of certain cancers 68, 69. High levels of CCL2 were consistent with increased macrophages infiltration, which promoted tumor development and metastasis 70, 71. Moreover, neutralization of CCL2 by anti-CCL2 in preclinical researches attenuated tumor burden and improved tumor immune microenvironment by impairing Th2 immunity and abrogating infiltration of TAMs 72, 73.

Hyperplasia and tumorous formation appeared to be connected with enhanced TAMs infiltration by prostaglandin E2 (PGE_2_), suggesting a modulator effect for PGE_2_ in TME 74. Generally, constitutively high CCL2 expression levels existed in tumors, such as melanoma, ovarian, lung and breast cancer, but not all types of cancer 75-78. In both tumor cells and macrophages, intracellular PGE_2_ signal was proved to induce the expression of CCL2 and directly guided macrophages chemotaxis to the tumor 79. However, downregulation of cellular reactive oxygen species (ROS) generation and cyclooxygenase-1 (COX-1) kinase activity were linked to mTORC2 inhibition, which significantly attenuated PGE2/CCL2-mediated chemotaxis and the number of macrophages 80-82. ROS regulates gene expression and may be involved in the production of chemokine in several cell types 83. There are multiple ROS sources in immune cells, including NADPH oxidases, mitochondria electron transportation, nitric-oxide synthases, and arachidonic acid metabolism 84, 85. However, in macrophages, COX-1-driven arachidonic acid metabolism is a significantly potent mediator for cell proliferation and the source of PGE_2_ 86. Thus, via ROS generation, mTORC2 was regarded as a modulatory node in PGE_2_-induced CCL2 expression and enhanced CCL2/CCR2 axis mediated macrophages chemotaxis 87, 88. Not surprisingly, consistent with its oxygen-sensing mechanism, hypoxia tensions reduced expression of CCL2 and suppressed macrophages migration and extension in TME 80. Furthermore, in esophageal cancer, TAMs were closely associated with the decreased number of CD8^+^ anti-tumor T cells. Accumulation of CCL2-mediated macrophages were negatively connected with CD8^+^ T cells amount and promoted tumor progress. However, CD8^+^ T cells antitumor efficacy recovered after CCR2 knockout 88. Therefore, diminishing the CCL2 and CCR2 interaction through the mTOR cascade may reduce TAMs recruitment and chemotaxis, subsequently promoting the anti-tumor effect of CD8^+^ T cells in TME. Through inhibiting monocytes infiltration and macrophages accumulation in TME, CCL2 intervention also suppressed the development of several types of tumor and promoted the better prognosis, including esophageal and breast cancers 71, 88. Together, mTOR signaling promotes the PEG-mediated CCL2 expression in both tumor cells and macrophages, thereby regulating macrophages chemotaxis to the tumor and acts as a potential target for anti-tumor immunotherapy.

**Figure 3:**
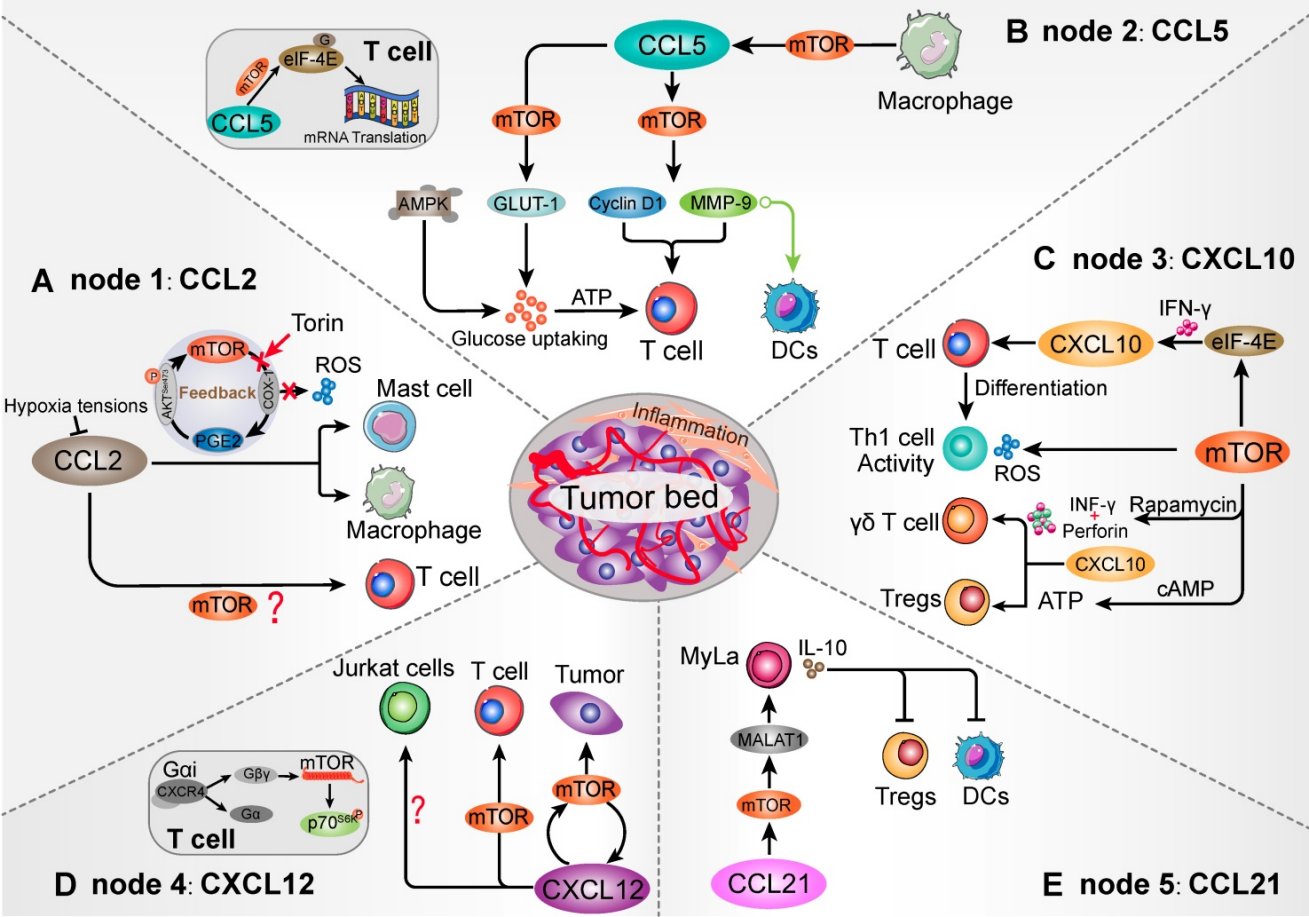
** Schematic diagram of the mTOR-related chemokines in immune cell chemotaxis**. Five nodes that can be targeted when mTOR influences the TME immune cells population. Homing into the tumor foci and migrating within the tumor environment, mTOR establishes contact with various immune cells that have been modulated via recognizable chemokines. **(A)** mTOR/PGE2 loop is involved in CCL2-mediated Mast cells and macrophages migration. However, whether mTOR modulates T cells by CCL2 remains to be further elucidated. **(B)** mTOR signaling in the macrophages intracellular affects the migration of CCL5-mediated T cells and DCs. As a downstream of mTOR, eIF-4E is not merely involved in T cell migration, but also affects CCL5-related mRNA translation by binding to 5'-cap mRNA structure in T cells, such as cyclin D1 and MMP-9. Moreover, mTOR-induced membrane protein GLUT1 shows the ability to promote glucose uptake and ATP generation to maintain T cells migration. **(C)** mTOR signaling acts as an upstream of CXCL10-mediated T cells, γδ T cells, as well as Th1 cells generation and activity. mTOR-mediated ATP generation contributes to Tregs accumulation and immunosuppressive activation, which may collaborate with CXCL10/CXCR3 interaction. **(D)** mTOR/CXCL12 cascade promotes T cells and tumor cells migration. The CXCL12/CXCR4 interaction could transform the conformation of CXCR4 into βγ-subunits, which can directly interact with mTOR carboxyl terminal. However, mTOR signaling is not involved in influencing the CXCL12-mediated Jurkat cells chemotaxis.** (E)** Intracellular mTOR cascade facilitates CCL21-medaited Myla cells migration and induces DCs and Tregs suppression. The mTOR activation can promote the expression of CCL21-associated MALAT1 in MyLa cells, which may influence Myla cells chemotaxis and IL-10 secretion. Increased IL-10 shows a potential to DCs and Tregs suppression.

As previous study, mTOR blocking in the mast cells can decrease PGE_2_-induced CCL2 production and chemotaxis 81. After the mTOR inhibitor Torin treatment, down-regulation of mTORC2 attenuated PGE_2_ function and CCL2 secretion 81. In a feedback loop, PGE2 was suggested to stimulate mTORC2 activation by increasing AKT (Ser473) phosphorylation. Moreover, mast cells expressed a variety of receptors that may contribute to the chemotaxis of mast cells, especially the CCR2 recognition function of CCL2 89, 90. Within the CCL2/CCR2 axis, IL-3^+^ stem cell factor (SCF) promoted mast cells migration in response to CCL2, which was positively connected with PI3K/mTOR cascade 90, 91. Furthermore, elevated CCR2 in mast cells showed effective antigen presentation and CD8^+^ T cells activation 92. Mast cell-derived leukotriene B4 (LTB4) was also required for CD8^+^ T lymphocytes recruitment in tumors 92. Herein, mTOR cascade might be an important signaling intermediary in CCL2-mediated mast cells chemotaxis and immune cells response (Figure 3A).

On the other hand, with the multifunctional activities of chemokines, expression of CCL2 had a positive role in TME and anti-tumor immune response. For example, tumor-derived CCL2 expression elicits primary T cells chemotaxis to sites of tumor foci and tumor regression in adoptively transferred cells (ATCs) therapy 93, 94. Transferred T cells showed a significant migration towards xenograft tumor in response to tumor-derived CCL2, whose transmigrate was directly mediated by CCR2 recognition. CCL2 directly manipulates T cell tropism toward tumor foci in a protective role, which is abolished by anti-CCL2 neutralizing treatment 95. Therefore, tumor regression related T cells dose not only depend on antigens recognition, but also are regulated by tumor-derived chemokines. However, whether mTOR cascade affects CCL2-mediated T cells chemotaxis in tumor remains to be further elucidated.

### CCR5/CCL5 signal

TILs expressing CCR5 are important for their infiltration in tumor foci 96. While, expression of CCR5 cognate ligands were correlated with anti-tumor immunity response in human melanoma, such as CCL3, CCL4 and CCL5 97, 98. Among which, chemokine C-C motif ligand 5 (CCL5) is the member of β-subtype chemokines, showing chemoattractive activity towards T cells, macrophages, DCs, and natural killer (NK) cells through the recognition of CCR5 58. Regulation of T-cell directional migration is a process that requires extracellular chemoattractant molecule signaling to active chemotaxis 99. Recently, estimation of TILs before immunotherapy was found to play an important role in immune checkpoint blocking effect in melanoma 100. In the context of adoptive T cell therapy, TILs activation and directional aggregation can contribute to the eradication of tumors via genetic and TME modifications 101, 102. It is now clear that CCL5 expression is positively correlated with TILs desertification, which orchestrates CD3^+^ T cells, CD4^+^ T cells and CD8^+^ T cells chemotaxis and immune response 20, 50, 78.

Mouton et al. utilized real-time microscopy and indicated that CCR5-positive Jurkat T cells showed a response to CCL5 forming sharp front and directional migration toward the chemokine aggregation region 103. In primary monocytes, mTOR signaling blocking was proved to suppress CCL5 secretion, thereby downregulating the amount of infiltrating T cells 104. Generally, activation of mTOR signaling promoted the function of two well-characterized downstream effectors in RNA and protein synthesis, such as p70 ribosomal S6 kinase (p70^S6K^) and eukaryotic initiation factor-4E-binding protein-1 (4E-BP1) phosphorylation 105. This cascade influenced T cells and DCs activation, proliferation and expansion in a macrophage-dependent manner (Figure 3B). In line with this, mTOR inhibition in macrophages directly reduced CCL5 generation and macrophages proliferation, which attenuated the chemotaxis of activated T cells 104, 106, 107. In which, translation efficiency of CCL5 was directly suppressed by 4E-BP1/2, and activation of 4E-BP1/2 produced lower CCL5 levels. The proportion and chemotaxis of activated T cells were associated with CCR5 expression and CCL5 recognition rate 107, 108. Thus, mTOR signaling contributes to regulate the chemotaxis of CCL5-responsive T cells by influencing the macrophages CCL5 mRNA expression. Moreover, Patel et al. indicated that mTOR inhibitors can significantly decrease CCL5 levels even after stimulation by neurotensin, a cytokine that can boost CCL5 gene expression and releasing 109. These indicated the priority role of macrophages mTOR signaling in CCL5 expression and biological function. Indeed, for efficiency of T cells, whose chemotaxis and adhesion are essential for effective anti-tumor immunoreactivity.

The multistep, efficient process of chemotaxis in immune cells not merely requires chemokines recognition, but also delivers the further modulatory signal to invoke cell migration. In the CD4^+^ T cells, CCL5-mediated chemotaxis and activity partially relied on intracellular mTOR signaling 58. After CCL5 is recognized by CCR5, it can stimulate the mTOR cascade and resolve the 4E-BP1 into eukaryotic initiation factor-4E (eIF-4E) 110. Subsequently, eIF-4E can interact with scaffold protein eIF-4G and form a translation initiation complex. CCL5 initiates eIF-4E complex in a sequential manner, which can facilitate ribosome to 5'-cap mRNA structure and promote the production of chemotaxis related proteins, such as cycloheximide, cyclin D1 and Matrix metalloprotein-9 (MM-9) 58, 111-113. Among these, CCL5-induced cycloheximide acted as the mediator of CD4^+^ T cells migration in a dose dependent manner. Consistent with this, an anti-MMP-9 antibody reduced CCL5-mediated migration of DCs 114. Notably, CCL5-induced mRNA translation in CD4^+^ T cells chemotaxis were significantly suppressed by mTOR inhibitor treatment 58. Herein, intracellular mTOR cascade is prime in CCL5-mediated T cells effective chemotaxis and related protein generation.

The metabolism and survival of effector T cells was tightly connected to glucose uptake 115. To facilitate T cells trafficking, chemokines also invoke diverse biological processes in TME (Figure 3B) 116. Energy metabolism is important for tumor immune cells to maintain housekeeping functions and T-cell activated status, increased metabolic was demanded to sustain the proliferation, growth, and immunizing potential 117. Moreover, cellular mTOR cascade has been proved to favor the anti-tumor effect and immune cells metabolic activity 118. A previous study indicated that the CCL5/mTOR cascade plays a pivotal role in energy generation to maintain high energy demands of activated T cells chemotaxis 119. In T cells, CCL5/CCR5-induced glucose uptake and ATP accumulation mainly depend on mTOR cascade manner, and mTOR signaling is necessary to promote intrinsic glucose transporter-1 (GLUT-1) to enhance glucose uptake. However, this process is not accompanied by surface levels of GLUT-1, suggesting that CCL5-stimulated glucose uptake mainly depends on mTOR signaling rather than transporter expression. Meanwhile, mTOR inhibition partly reduced CCL5-mediated glucose uptake compared to glycolysis and ATP production inhibitors 120. In this aspect, AMPK loop was partly required for ATP generation to sustain CCL5-mediated T cells activation and chemotaxis 119. Therefore, CCL5 may simultaneously induce both mTOR and AMPK cascade to ensure the T cells effective chemotaxis. Taken together, these studies suggest that mTOR signaling has an ameliorative role in CCL5-mediated efficient T cells migration in TME.

### CXCR3/CXCL10 signal

In the TME, CXCR3 is expressed in T cells, whose ligands (CXCL9, CXCL10 and CXCL11) facilitate effective recruitment of CXCR3^+^ T cells to tumor foci 121. Among these, CXCL10, a member of the CXC chemokine subfamily, also known as INF γ-induced protein 10 kDa (IP-10) 122. CXCL10 is strongly induced by IFN-γ and IFN-α/β, and weakly induced by TNFα 123, 124. Meanwhile, IFN-γ induced CXCL10 was primarily secreted by monocytes, macrophages, eosinophils, and endothelial cells 125. Comparison of tumor tissues and peripheral blood demonstrated that CXCL10/CXCR3 chemokine axis serves as a potent chemoattractant for T lymphocytes in TME 126. In addition, CXCL10 is important for recruitment of T lymphocytes such as CD8^+^ T cells in the tumor tissues, and is positively correlated with increased survival and favorable prognosis 127. Up-regulated CXCL10 expression contributes to lower tumor burden and malignancy, serving as a positive determinant of anti-tumor immune responses, although its exact underlying immunoreaction mechanism in TME yet to be clear 128.

The CXCL10/CXCR3 interaction has been demonstrated to regulate immune cells chemotaxis and activation in TME, and improve the efficacy of cancer immunotherapy (Figure 3C) 129. Recently, mTOR-generated signal was suggested to play a positive role in the IFN-related biological roles, including T cells immune response and tumor regression 130. As evidenced by the negative regulatory properties of 4E-BP1 upon embryonic fibroblasts IFN-specific CXCL10 translation and protein synthesis, mTOR inhibition exerts an important regulatory effect on the IFN responses 131, 132. The mTOR blocking makes the sequential dissociation of 4E-BP1 to eIF-4E, subsequently promoting the CXCL10 transcription in response to IFN signal, especially INF γ. When the vascular endothelial is damaged, CXCL10 attracted by CXCR3^+^ T cells was accompanied with mTOR signaling stimulation. CXCR3^+^ T cells recruitment and ROS generation were directly associated with mTOR-dependent manner, which promoted the activity of Th1-mediated immunity 132. The T cells CXCL10/CXCR3 interaction also invoked the great interest in the differentiation of naive T cells to Th1 and the chemotaxis of activated effector T cells to tumor focal sites, which were mediated by INF γ 133. Therefore, enhancing the level of CXCL10 in tumor cell or TME could promote a strong T cells anti-tumor immunity, inhibiting tumor cells progress and angiogenesis 134. Herein, the mTOR cascade in both tumor cells and T cells were likely to associated with CXCL10-mediated T cells chemotaxis and anti-tumor effect. Furthermore, in macrophages, the mTOR cascade controlled the CXCL10 mRNAs translation through 4E-BP1/2 node, which subsequently affected the T cells chemotaxis 107. Relying on translational repression of 4E-BP1/2 by CXCL10, mTOR inhibition can activate 4E-BP1/2 and reduce CXCL10 generation. In line with these evidences, the stimulation of mTOR cascade may enhance CXCL10-mediated T cells chemotaxis and anti-tumor immune responses in TME.

In another aspect, for the γδ T cells, tumor-intrinsic mTOR inhibition promotes the epidermal recruitment of CXCL10-mediated CXCR3^+^ γδ T-cell and shows anti-tumor effect 135. However, the mechanisms between mTOR and CXCR3^+^ γδ T-cell are unchecked. As report, the perforin induced by mTOR-blocking can synergize with INF-γ, thereby promoting the killing function of CXCL10-mediated γδ T cells in tumor 135. Importantly, the biological functions of the heterogeneous subsets of γδ T cells are essential for various immune responses and immunopathology, such as shaping the T-cell repertoire, distinct kinetics, and response to specific pathogens 136. As Jean-Jacques report, more than 250 patients with hematological malignancies and solid tumors received γδ T-cell-based cancer immunotherapy, which regulated immunosuppressive functions mainly through the shaping of γδ T cell activation 137. Tumor-infiltrating γδ T cells were widely detected in tumors and interacted with tumor cells, and up-regulated γδ T cells infiltration subsequently prevented tumor progression 138. Moreover, the migration of γδ T cells in pyometra samples was suggested to be associated with up-regulation of CXCL10 139. Together, in TME, although the mTOR cascade might be involved in CXCL10-mediated γδ T cells chemotaxis, the mechanisms still need to be further illustrated.

One interesting find is that mTOR cascade plays a critical role in Tregs fate determination. In the mouse model, mTOR activation contributes to Tregs accumulation and immunosuppressive activation 140-142. In tumor, Tregs activation decreases various cell populations via multiple mechanisms, which is a barrier for effective antitumor immunity, such as tolerance or rejection 143. Moreover, dynamic Tregs have high metabolic state and ATP demanded, and mTOR signaling was positively connected with Tregs migration 144. Among which, cAMP participates in the mTOR-mediated metabolic activation, resulting in an enhanced ATP generation. Besides, CXCR3^+^ Tregs showed immunosuppressive and carcinogenic effects in response to CXCL10, which was connected with Tregs chemotaxis 145. Therefore, mTOR activation might be also associated with CXCR3^+^ Tregs intracellular metabolic and energy status for chemotaxis.

### CXCR4/CXCL12 signal

The binding of CXCL12 to CXCR4 initiates intracellular signaling, which can cause a series of responses like chemotaxis, proliferation, and calcium afflux 146. Two major isoforms of CXCL12 are divided into α/β subtypes and share agonist potency to cognate receptor CXCR4 147. The α-isoform is involved in many local tissue-specific physiological processes, while the β-isoform is associated with peripheral blood cells infiltration. In renal cyst (RC) cells, the mTOR cascade acts as the downstream of CXCL12/CXCR4 axis, and CXCR4-mediated CXCL2 recognition can selectively active the mTOR signaling to enhance RC cells proliferation 148. This process may also exist in human T cells. Moreover, mTOR inhibition directly suppressed CXCL12-mediated primary resting T cells and CEM cell (T-cell leukemia), because CXCL12 directly utilized the intracellular mTOR cascade in T cells chemotaxis 62 (Figure 3D). As a mTOR downstream effector, p70^S6K1^ has an important role in mTOR function in T cells chemotaxis. Moreover, p70^S6K1^ and mTOR blocking can attenuate the CXCL12-induced T cells chemotaxis. CXCL12 recognizes and alters CXCR4 conformation by favoring conversion of Gαi protein into α- and βγ-subunits 146. Gβγ heterodimers can directly interact with the intrinsic mTOR carboxyl terminal region and promote phosphorylation of p70^S6K^ 149. Therefore, mTOR signaling is positively regulated by direct interaction of Gβγ, which is involved in CXCL12-mediated T cells chemotaxis and plays an important role in host immune surveillance. In the immune system, CXCR4 is highly expressed by monocytes, B cells, and naive T cells in peripheral blood 150, 151. It is not surprising that dysregulated CXCL12/CXCR4 axis can influence cancer cells killing effect and activities of multiple immune cells and chemotaxis in TME 152, 153.

Immune cells migration is tightly correlated to disease progress in both innate and adaptive immune environment. With a gradient of CXCL12/CXCR4 axis to mTOR signaling, immune cells may lead to a directional chemotaxis in TME, such as CD4^+^ T cells and DCs along with the CXCR4 expression 154, 155. CXCL12 mainly acts as an effective chemoattractant for T cells, and modulates T cells chemotaxis under various pathophysiological conditions, including tumorigenesis and inflammation 156, 157. In the Jurkat cells, chemokine receptor CXCR4 response to CXCL12 is required for cells migration 158, 159. However, CXCL12-mediated Jurkat cells chemotaxis is in contrast to the phenomenon of human primary T cells that cannot be abrogated by mTOR inhibition 157. Application of mTOR selective inhibitor rapamycin in Jurkat cells also has no significant effect on migration gene set. Of course, alteration at the mRNA level alone cannot fully predict CXCL12-mediated chemotaxis. Moreover, two other mTOR p70^S6K^ inhibitors (lindane and DON) also fail to affect the CXCL12-related Jurkat cells migration 157, 160.Therefore, unlike other primary T cells, mTOR signaling dose not influence the CXCL12-mediated Jurkat cells chemotaxis.

Intriguingly, CXCL12/CXCR4 interaction induced mTOR signaling not only influences the immune cells chemotaxis, but also participates in tumor cells migration, such as gastric, pancreatic and renal cancer cells 161-163. mTOR signaling and CXCL12/CXCR4 axis have been reared as a positive feedback loop to influence the tumor migration and CXCL12 secretion. Due to the 5'-cap-dependent translation, mTOR inhibition decreased the secretion of CXCL12 and the expression of receptor CXCR4, which subsequently blocked the CXCL12-triggered tumor cells chemotaxis and malignant 161, 163. Moreover, as a feedback, attenuated CXCL12/CXCR4 interaction will reduce the activity of Gαi protein and decrease the activation of mTOR signaling. Here, targeting the mTOR pathway may prevent cancer metastasis driven by CXCL12/CXCR4 interaction 161. Taken together, various CXCL12-mediated T cells and tumor cells chemotaxis were connected with mTOR signaling, but mTOR interventional therapy plays a pros or cons role in different conditions of TME, and it is unclear.

### CCR7/CCL21 signal

CC-chemokine receptor 7 (CCR7) is expressed in various subtype of T cells, and recognized by its ligands are essentially involved in the migration of T cells, Tregs and DCs in immunity environment 164. CCL19 and CCL21 are the sole ligands for the CCR7 receptor. The CCL21/CCR7 interaction is positively correlated with T cells chemotaxis in shaping TME, which can enhance the homing of CCR7-expressing T cells and response to systemic anti-tumor immunotherapy 165-167. On the contrary, chemokine-mediated abnormal T lymphocytes migration contributed to tumor progress. For example, CCR7 positive mycosis fungoides cells (CCR7^+^ MyLa) migration can be stimulated by its agonist CCL21 in a mTOR-dependent manner 168. The migration and activity of MyLa cells and the phosphorylation of downstream p70^S6K^ were significantly up-regulated after CCL21 treatment (Figure 3E). Meanwhile, mTOR inhibition suppressed CCL21-induced MyLa cells migration, rather than Peripheral Blood Mononuclear Cells (PBMCs) 168. These evidences indicated that mTOR activity was directly involved in CCL21-mediated MyLa cells chemotaxis.

Mycosis fungoides (MF) is the most common form of cutaneous T-cell lymphoma characterized by malignancy of resident T cells, which is indolent but can potentially progress to leukemia 169, 170. Following the occurrence of malignant T cells in skin, the release of cytokines and chemokines is vital for the initiation or duration of the MF lesion 171, 172. The activated MF immune environment increases interleukin-10 (IL-10) and transforming growth factor β (TGF-β) levels to regulate normal host T cells, both of which can contribute to the inhibition of anti-tumor immunity 149, 152. As a novel regulator in immunity, metastasis-associated lung adenocarcinoma transcript 1 (MALAT1) has highly selective IL10 secretion, which can contribute to the tolerogenic DCs and antigen-specific regulatory T cells suppression, thereby attenuating innate immune response 173. Hong et al. indicated that MALAT1 expression acts as the downstream target of mTOR and promotes CCL21-mediated MyLa cells migration (Figure 3E) 60. Furthermore, MyLa-expressed MALAT1 was selectively increased in MF site. Intriguingly, mTOR inhibition suppressed both MALAT1 expression and CCL21-induced MyLa cells migration. However, MALAT1 knockdown only inhibited the MyLa cells migration rather than CCL21-induced mTOR activity 60. Therefore, both the mTOR or MALAT1 could be regard as a potential target to inhibit the MyLa cells chemotaxis response to CCL21.

## Conclusions and future perspectives

Here, we have provided an outline of mTOR function in chemokines-related reactions in both immune or tumor cells, as well as their cascade gene activation. This modulator node is crucially involved in efficient immune cells migration. Emerging evidences highlight the importance of immune cells migration and infiltration in TME, especially T lymphocytes and macrophages that determine the outcomes of cancer immunotherapy. The roles of mTOR signaling in regulation of chemokine-mediated immune cells are quite diverse, and include promotion of T cells accumulation and eradication in tumor foci, as well as boosting tumor development and immune evasion. The hijacking of this pathway by tumor cells for malignant or immune evasion may present an opportunity to develop T cell-based immunotherapy. Likewise, chemical inhibition of the mTOR signaling greatly facilitates enhancement or suppression of chemokine-mediated T cells chemotaxis in tumor environment or inflammation. These avenues will undoubtedly provide new frontiers for innovations towards efficient immunotherapy.

In the current studies, there are also critical evidences elucidating how dysregulation of mTOR signaling facilitates tumorigenesis. Although the mTOR cascade and chemokine/receptor axis can either present positive or negative for antitumoral roles in regulating immune cells or tumor cells migration, which of its roles determines the outcome is still remains an open question. Future studies also need to explore pros or cons of mTOR intervention in regulating biological environment of chemokine-related immune cells migration or anti-tumor function. Does mTOR guide energy metabolism or other chemokines in selective immune cells towards cancer cells? In this regard, it will be interesting to determine the diverse abilities of mTOR to regulate immune cell chemotaxis and tumor eradication.

## Figures and Tables

**Table 1:** mTOR inhibitors under clinical investigation.

Drug	Phase	Clinical trail	Condition	Target	Description
Rapamycin	Phase Ⅱ	NCT02874924	Age-associated pathologies/Tumor	T cells	Enhancing immunity
	Phase Ⅱ	NCT01620307	H1N1 pneumonia	T cells and B cells	Improves hyperactive environment
	Phase Ⅳ	NCT02990312	Kidney Transplant	CCR5 density	Enhancing immunity
	Phase Ⅰ	NCT01522820	Solid Tumors	T cells	Enhancing anti-tumor immunity
	Phase Ⅰ	NCT02833506	Ovarian cancer	T cells	Induced anti-tumor immunity
	Phase Ⅰ	NCT01536054	Ovarian cancer	T cells	Induced anti-tumor immunity
	Phase Ⅰ/Ⅱ	NCT03662412	Pancreatic cancer	T cells	Reverses the immune suppressive microenvironment; T lymphocytes activation and proliferation
Everolimus	Phase Ⅱ	NCT01665768	Lymphoma	B cells	Reduce the frequency of circulating cancer cells
	Phase Ⅱ	NCT01234974	Myeloma	B cells	Inhibit cell growth
	Phase Ⅱ	NCT01637090	Lymphoma	T cells	Inhibit malignant T cells
	~~~	NCT02441543	Solid Tumors	T-cell and NK cells	Cells phenotype and dynamics
	~~~	NCT03955172	Kidney Transplanted	NK cells	Prevent Rejection
Azithromycin	Phase Ⅱ	NCT04020380	Pulmonary Sarcoidosis	T cells and macrophages	Reduce inflammatory cytokine production

**Table 2:** mTOR-associated chemokines in cancer and pathological condition.

Category	Macropha	Th2	T cell	γδ T cell	Tregs	Jurkat T-cell	DCs	NK cell	Th1	CTLs	MyLa	B cells	References
Colon cancer	CCL2		CXCL10										68, 69, 138
Breast cancer	CCL2		CXCL12										71, 174
Stomach cancer	CCL2												79
Ovarian cancer	CCL2		CCL5/CXCL10										22, 80, 127, 128
Glioma			CCL2		CCL2								93, 175
Renal cell cancer			CCL2/CXCL10										93, 133
Melanoma			CCL2/CCL21/CXCL12	CCL2				CXCL10		CXCL12			94, 95, 152, 165, 176-178
Leukemia (APL)						CCL5							103
Esophageal			CCL5/CXCL10										126
Atheroma									CCL10				176
Lung fibrosis				CXCL10									179
Leukemia			CXCL12										62
Cutaneous											CCL21		60, 168, 171, 180
Wound healing		CCL2											73
Skin tumors				CXCL10									135
Inflammation	CCL5		CXCL12				CCL5					CXCL12	62, 107, 114, 150, 156

## Abbreviations

mTOR: Mammalian target of rapamycin
TME: Tumor microenvironment
mTORC1: mTOR protein complexes 1
PI3K: Phosphoinositide 3-kinase
Th1: T helper type 1
Th2: T helper type 2
Tfh: T follicular helper
APCs: Antigen presenting cells
RTK: Receptor tyrosine kinases
PIP2: Phosphatidylinositol-4,5-bisphosphate
PIP3: Phosphatidylinositol-3,4,5-trisphosphate
PH: Pleckstrin homology
DCs: Dendritic cells
TCR: T-cell receptor
Tregs: Regulatory T cells
RHEB: RAS homolog enriched in brain
CCL: Chemokine C-C motif ligand
CXCL: Chemokine C-X-C motif ligand
CCR: CC chemokine receptor
CXCR: C-X-C chemokine receptor
TAMs: Tumor-associated macrophages
PGE2: Prostaglandin E 2
ROS: Reactive oxygen species
COX-1: Cyclooxygenase-1
NADPH: Nicotinamide adenine dinucleotide phosphate
SCF: Stem cell factor
LTB4: Leukotriene B4
ATCs: Adoptively transferred cells
TILs: Tumor infiltrating lymphocytes
p70S6K: p70 ribosomal S6 kinase
4E-BP1: Eukaryotic initiation factor-4E-binding protein-1
eIF-4E: Eukaryotic initiation factor-4E
TSC1: TSC complex subunit 1
MMP-9: Matrix metalloprotein-9
GLUT-1: Glucose transporter-1
IP-10: INF γ-induced protein 10 kDa
RC: Renal cyst
MF: Mycosis fungoides
MyLa: Mycosis fungoides cells
TGF-β: Transforming growth factor β
MALAT1: Metastasis-associated lung adenocarcinoma transcript 1
